# MicroRNAs as Key Regulators in the Progression of Metabolic Dysfunction-Associated Steatotic Liver Disease: A Bioinformatics Analysis

**DOI:** 10.3390/biomedicines14010120

**Published:** 2026-01-07

**Authors:** Claudriana Locatelli, Karine Luz, Sergio Fallone de Andrade, Emyr Hiago Bellaver, Rosana Claudio Silva Ogoshi, Ariana Centa, João Paulo Assolini, Gustavo Colombo Dal Pont, Tania Beatriz Creczynski-Pasa

**Affiliations:** 1 Graduate Program in Development and Society, Alto Vale do Rio do Peixe University, Caçador 89500-199, Santa Catarina, Brazil; karine.luz@uniarp.edu.br (K.L.); rosana.ogoshi@uniarp.edu.br (R.C.S.O.); ariana.centa@uniarp.edu.br (A.C.); joao.assolini@uniarp.edu.br (J.P.A.); gustavo.colombo@uniarp.edu.br (G.C.D.P.); 2 Laboratory of Translational Research in Health and Laboratory of Biosaude, Alto Vale do Rio do Peixe University, Caçador 89500-199, Santa Catarina, Brazil; 3 Research Institute for Medicines (iMed.ULisboa), Faculty of Pharmacy, Universidade de Lisboa, 1649-003 Lisbon, Portugal; sfallone@ff.ulisboa.pt; 4 Department of Pharmacy, Pharmacology and Health Technologies, Faculty of Pharmacy, Universidade de Lisboa, Av. Prof. Gama Pinto, 1649-003 Lisbon, Portugal; 5 Center for Health Science, Alto Vale do Rio do Peixe University, Caçador 89500-199, Santa Catarina, Brazil; emyr@uniarp.edu.br; 6 Department of Pharmaceutical Sciences, Federal University of Santa Catarina, Florianópolis 88040-900, Santa Catarina, Brazil; tania.pasa@ufsc.br

**Keywords:** epigenetic, regulation, hepatic inflammation, insulin resistance, liver fibrosis, microRNA-122, microRNA-29a, miRNA target prediction

## Abstract

**Background:** Metabolic dysfunction-associated steatotic liver disease (MASLD), formerly known as non-alcoholic fatty liver disease, is a highly prevalent hepatic condition closely linked to metabolic syndrome (MetS). Epigenetic regulators such as microRNAs (miRNAs) have emerged as critical modulators of the molecular pathways underlying MASLD pathogenesis, offering new perspectives for non-invasive diagnosis and targeted therapy. This study aimed to identify and characterize target genes and pathways regulated by two key hepatic miRNAs, namely miR-122 and miR-29a, through a comprehensive in silico bioinformatics approach, to better understand their functional roles in MASLD and MetS. **Methods:** Target genes of miR-122 and miR-29a were predicted using three databases (TargetScan, DIANA-microT-CDS, and miRWalk), and those identified by at least two databases were selected for downstream analyses. Functional enrichment was performed using Gene Ontology and KEGG pathway analysis. Gene networks and biological process maps were constructed using Metascape, clusterProfiler and Cytoscape. **Results:** miR-122 was found to negatively regulate genes involved in lipid metabolism, insulin signaling, and inflammatory pathways, including *PPARGC1A*, *PPARA*, *LPL*, *TLR4*, and *HMGCR*, contributing to insulin resistance and liver dysfunction. By contrast, miR-29a demonstrated potential hepatoprotective effects by targeting *LEP*, *INSR*, *IL13*, and *IL18*, enhancing insulin sensitivity and reducing fibrogenic activity. Enrichment analysis revealed strong associations with biological processes, such as STAT phosphorylation, lipid homeostasis, and inflammatory signaling, as well as associations with cellular components, including lipoproteins and plasma membranes. miR-122 and miR-29a exhibit opposing regulatory functions in MASLD pathogenesis. Whereas miR-122 is associated with disease progression, miR-29a acts protectively. These miRNAs may serve as promising biomarkers and therapeutic targets in MASLD and related metabolic conditions. Further validation through experimental and clinical studies is warranted.

## 1. Introduction

Metabolic dysfunction-associated steatotic liver disease (MASLD), previously known as non-alcoholic fatty liver disease, has become the most prevalent chronic liver disease worldwide. Closely linked to metabolic syndrome (MetS), it has insulin resistance and visceral adiposity as its core components [1,2]. The disease currently affects approximately 25% of the global population and encompasses a spectrum ranging from hepatic steatosis to steatohepatitis and fibrosis, with potential progression to hepatocellular carcinoma (HCC) [3,4].

The central role of insulin resistance in the pathophysiology of MASLD is well established: it contributes to hepatic lipid accumulation, inflammation, and fibrogenesis [5,6]. Despite advances in clinical recognition, the molecular mechanisms underlying MASLD remain incompletely elucidated, and there are no approved pharmacological therapies targeting its pathogenesis [7].

Visceral obesity is strongly associated with increased liver fat, independent of total body weight [8]. This relationship reinforces the view that MASLD is not a mere consequence of general obesity, but rather a hepatic manifestation of MetS, with insulin resistance forming the central link between these conditions [9]. Visceral adipose tissue dysfunction aggravates insulin resistance and is directly associated with the severity of MASLD, even in individuals without apparent obesity [6,8].

The diagnosis of MASLD is often incidental, based on elevated liver enzyme levels or hepatic steatosis detected by imaging studies. The condition is unrelated to excessive alcohol use [7]. Although noninvasive methods are being developed to assess liver fibrosis and inflammation, liver biopsy is considered the gold standard for the diagnosis and staging of MASLD [10]. Lifestyle modification, including weight loss and a healthy diet, remains the mainstay of treatment for MASLD [11].

MicroRNAs (miRNAs) have emerged as promising molecular regulators in metabolic liver diseases. These small non-coding RNAs modulate gene expression post-transcriptionally and are involved in critical pathways, such as lipogenesis, inflammation, mitochondrial homeostasis, and fibrosis [12,13,14]. miR-122, the most abundant hepatic miRNA, and miR-29a, associated with oxidative stress modulation, have been consistently implicated in MASLD and related metabolic conditions [15,16].

With the growing availability of predictive and functional annotation tools, bioinformatics offers a powerful approach to map miRNA–gene interactions, prioritize molecular targets, and understand disease-associated signaling networks. However, the application of integrative bioinformatics analyses to key miRNAs, such as miR-122 and miR-29a, is still underexplored in MASLD research.

The current study performed a bioinformatics analysis to identify potential molecular targets and metabolic pathways regulated by miR-122 and miR-29a, contributing to our understanding of the role of these miRNAs in the pathophysiology of MASLD, particularly when associated with MetS. The aims were to predict the target genes of miR-122 and miR-29a, explore molecular interactions, and identify enriched metabolic pathways relevant to MASLD pathogenesis. The choice of these miRNAs is based on their well-documented functions in the regulation of key processes in MASLD progression, such as inflammation, liver fibrosis, and lipid metabolism. Studies support the clinical use of miRNAs as non-invasive biomarkers and promising therapeutic targets in the management of MASLD. In view of these considerations, this investigation sought to answer the following research question: What are the main molecular targets and metabolic pathways modulated by miR-122 and miR-29a in MASLD associated with MetS?

## 2. Materials and Methods

A comprehensive in silico bioinformatics analysis was performed to elucidate the role of miRNAs in the progression of MASLD associated with MetS. Based on existing literature, miR-122 and miR-29a were selected for their key regulatory functions in lipid metabolism, insulin resistance, and hepatic inflammation.

### In Silico Analysis of miRNAs and Genes Related to MASLD and MetS

The analysis included four stages: disease-associated gene retrieval, target gene prediction, functional enrichment analysis, and interaction network construction. Initially, the MalaCards platform (https://www.malacards.org/; version 5.20 accessed on 20 July 2024) was used to explore the disease profile of MASLD. MalaCards integrates data on human diseases, including genes, pathways, and biomarkers. The search was conducted using the terms “metabolic dysfunction-associated steatotic liver disease,” enabling the identification of MASLD-associated diseases, genes, and miRNAs. The top 10 comorbid conditions were selected based on co-occurrence frequencies and relevance scores, facilitating the construction of a disease network and the identification of key regulatory elements [17]. All data from MalaCards used in this study were retrieved between May and July 2024 using database version 5.20. The MalaCards platform is continuously updated, and since data extraction the database has progressed to version 5.26, which includes substantial revisions to disease annotations, relevance scores, and gene–disease associations. Therefore, numerical differences between the values reported here and the current MalaCards interface reflect changes introduced in subsequent updates.

Target gene prediction for miR-29a and miR-122 was conducted using three validated databases: TargetScan version 8.0 (https://www.targetscan.org/; accessed on 31 August 2024), which considers evolutionary conservation and contextual scoring; DIANA-microT-CDS (http://diana.imis.athena-innovation.gr; accessed on 31 October 2024) [18], which incorporates both 3′UTR and coding DNA sequence (CDS) regions; and miRWalk version 3.0 (http://mirwalk.umm.uni-heidelberg.de/; accessed on 31 October 2024), which integrates multiple predictive tools. For enhanced reliability, only genes predicted by at least two databases were included for further analysis.

Initially, all raw predictions were retrieved from the three platforms. A consensus list was generated by retaining only genes predicted by at least two databases. This consensus set was subsequently refined by applying reliability thresholds, namely: TargetScan context++ score < −0.2 and DIANA-microT score > 0.8. The resulting high-confidence genes were then subjected to disease-association annotation using the miRWalk platform, and only these final curated genes were forwarded to downstream enrichment and network analyses.

Subsequently, functional enrichment analysis was performed to categorize the predicted genes by biological process, molecular function, and metabolic pathway involvement. Gene Ontology [19] and Kyoto Encyclopedia of Genes and Genomes (KEGG) [20] analyses were conducted using Metascape (https://metascape.org/; accessed on 31 October 2024) and clusterProfiler [21] (https://www.bioinformatics.com.cn/SRplot; accessed on 31 October 2024), with a statistical threshold of *p* < 0.05 and false discovery rate (FDR) adjustment. Pathway visualization was carried out using the Pathview tool [22] (https://github.com/datapplab/pathview; accessed on 31 October 2024). Because enrichment algorithms evaluate pathway overrepresentation independently of the direction of regulation, all positively and negatively regulated targets were analyzed together to provide an integrated view of the biological pathways influenced by each miRNA.

For analysis of gene connectivity and biological interactions, interaction networks were constructed using Cytoscape [23] (https://cytoscape.org/; accessed on 31 October 2024) and Metascape [24] (https://metascape.org/gp/index.html; accessed on 31 October 2024). Network enrichment was filtered by statistical significance (*p* < 0.05 and adjusted FDR), allowing the identification of functionally relevant modules and hub genes regulated by the selected miRNAs. This integrative bioinformatics workflow enabled the mapping of molecular pathways and target genes modulated by miR-122 and miR-29a, offering mechanistic insights into their role in the metabolic and fibrogenic processes underlying MASLD.

## 3. Results

### 3.1. Disease-Gene-miRNA Network Analysis for MASLD and MetS

The MalaCards platform was used to investigate the molecular landscape underlying MASLD in the context of MetS. This comprehensive resource enabled the identification of genes, miRNAs, and comorbidities associated with MASLD, facilitating the generation of biologically grounded hypotheses for further in silico analyses. The platform provided an integrated view of the molecular network involved in the MASLD–MetS interplay, contributing to the identification of potential diagnostic biomarkers and therapeutic targets.

According to data extracted from MalaCards, key clinical risk factors for MASLD include abnormal body mass index, elevated glucose levels, hypertension, hypertriglyceridemia, and reduced HDL-C levels, all of which are hallmark features of MetS. Genomic profiling revealed that MASLD is associated with 108 genes and 340 diseases, underscoring its systemic and multifactorial nature. In comparison, MetS was linked to 1148 genes and 1125 associated conditions, reflecting its broad impact on metabolic regulation and disease progression.

**Figure 1:**
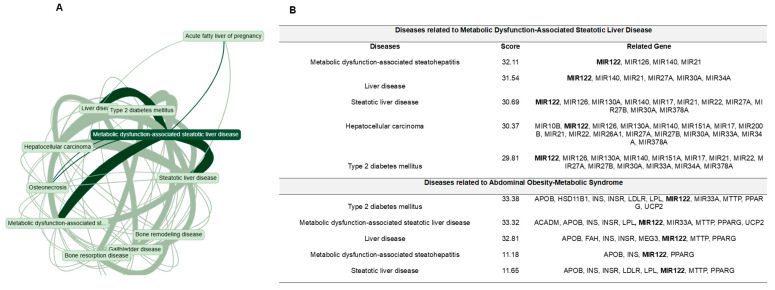
Association network of metabolic dysfunction-associated steatotic liver disease (MASLD), other metabolic conditions, and related genes. Lines indicate relationships identified in biomedical literature, with their thickness proportional to the strength of associations. (**A**) Disease–disease association network automatically generated by MalaCards. The network displays the ten diseases with the highest MalaCards association strength, an integrated metric derived from relevance score, elite evidence level, gene–disease associations, phenotypic similarity, and literature co-occurrence. (**B**) Gene association table retrieved from the MalaCards MASLD gene panel and from the gene panels of the comorbid diseases identified in panel (**A**). Scores correspond to MalaCards disease relevance scores.

Figure 1A illustrates the multifactorial overlap among MASLD, MetS, type 2 diabetes mellitus (T2DM), HCC, bone disorders, and biliary tract diseases. Figure 1B highlights the role of several miRNAs, such as miR-122, miR-21, miR-33a, and miR-126, as key regulators of hepatic homeostasis, inflammation, and lipid metabolism.

**Figure 2:**
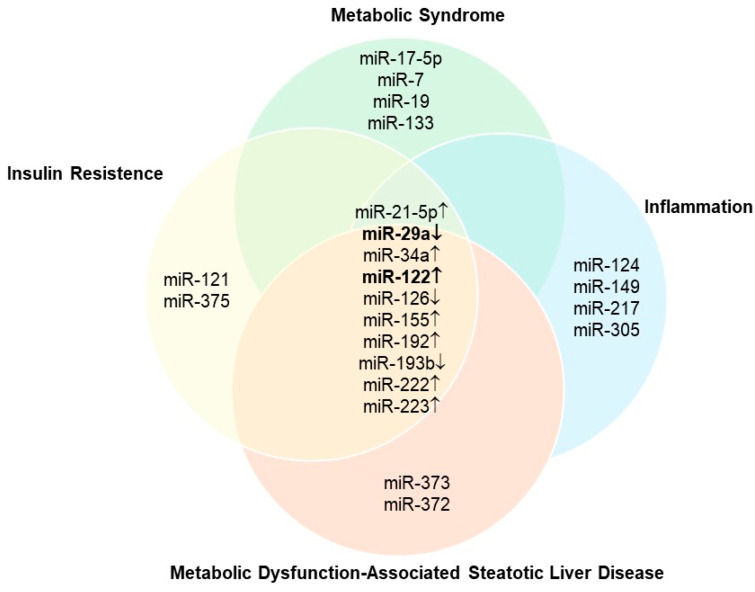
Venn diagram representing the differential expression of microRNAs (miRNAs) across various biological conditions. miRNAs in the center of the diagram, including miR-122 (↑) and miR-29a (↓), represent regulatory molecules common to diverse pathophysiological contexts, suggesting a central role in metabolism and inflammatory pathways. Arrows indicate the direction of regulation: (↑) increased expression and (↓) decreased expression.

A Venn diagram was constructed using MalaCards data and supporting literature (Figure 2) to represent miRNAs dysregulated in MASLD and MetS and their involvement in central pathological features, such as insulin resistance and systemic inflammation.

### 3.2. miRNA Target Prediction and Selection of Candidate Genes

Table 1 summarizes the number of final curated genes predicted as targets of miR-122 and miR-29a across three major metabolic disorders, namely T2DM, MetS, and MASLD, following the multi-step filtering described in the Methods Section. The table displays only the high-confidence genes that met all prediction and reliability criteria and were subsequently annotated for disease associations using miRWalk. This approach highlights the overlapping regulatory potential of these miRNAs in key metabolic and inflammatory pathways.

The number and identity of predicted target genes differed according to condition and miRNA strand (3p and 5p), reflecting the context-dependent regulatory activity of the analyzed miRNAs. Notably, miR-29a-5p and miR-122-5p exhibited the highest number of targets, particularly in MASLD, reinforcing their potential as central epigenetic regulators.

As detailed in the Methods Section, target prediction was initially performed using TargetScan v8.0, DIANA-microT-CDS, and miRWalk v3.0. Only genes identified by at least two databases were retained, and this consensus list was further refined using reliability thresholds (TargetScan context++ score < −0.2, (ii) DIANA-microT score > 0.8). After this filtering process, the remaining genes were annotated for disease associations using the miRWalk platform, resulting in the final curated set presented in Table 1.

Among them, *LEP*, *LPL*, *PPARGC1A*, *HMGCR*, *INSR*, *IGF1*, *CCL5*, *TLR4*, *IL13*, and *IL18* were consistently identified and selected for downstream enrichment analysis. These genes are involved in lipid and glucose metabolism, insulin signaling, adipokine regulation, and chronic inflammation. These processes are implicated in the pathogenesis and progression of MASLD and MetS.

Integrative analysis provided a foundational set of candidate genes to be explored in subsequent functional enrichment and pathway analyses, offering insights into the molecular networks modulated by miR-122 and miR-29a in MASLD and its associated metabolic comorbidities.

### 3.3. Functional Enrichment Analysis of miR-29a and miR-122 Target Genes

The current study proposed an innovative approach, focused on the application of bioinformatics tools for the prediction of genes regulated by miR-29a and miR-122 in models associated with MASLD. This strategy allowed the construction of regulatory networks and the identification of molecular pathways potentially implicated in the pathophysiology of MASLD, representing a methodological advance and a promising step toward identifying new diagnostic and therapeutic targets mediated by miRNAs.

**Figure 3:**
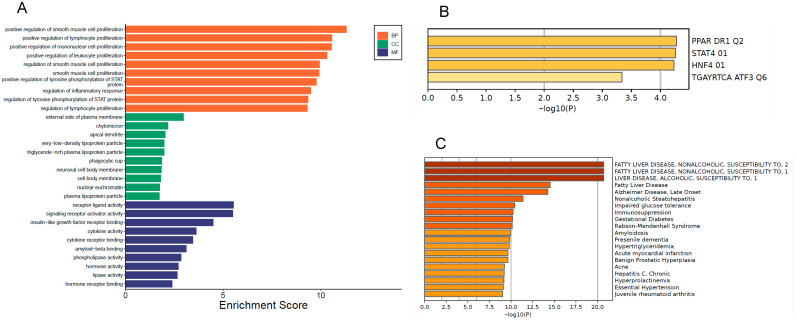
Functional enrichment analysis and association with metabolic diseases. (**A**) Gene Ontology (GO) enrichment for *Biological Processes* (BP, orange), *Cellular Components* (CC, green), and *Molecular Functions* (MF, blue). Bars represent enrichment scores (−log10 *p*-value). (**B**) Transcription factor (TF) enrichment analysis identifying upstream TFs predicted to regulate the miRNA-target gene set. The top TFs include PPAR DR1 Q2, STAT4, and HNF4. (**C**) Disease-association enrichment analysis showing conditions most strongly linked to the miRNA-regulated genes, including MASLD, T2DM, insulin resistance, hypertriglyceridemia, and related metabolic and inflammatory disorders. Bars represent −log10 (*p*-value).

Figure 3 presents the functional enrichment analysis of genes predicted to be regulated by miR-29a and miR-122, highlighting enriched categories within biological processes, cellular components, and molecular functions. Additionally, the analysis explores transcription factors potentially involved in the regulation of these genes and their association with key metabolic diseases, including MASLD, T2DM, and insulin resistance.

Among the enriched biological processes (Figure 3A), notable pathways included the positive regulation of smooth muscle cell, lymphocyte, and leukocyte proliferation, as well as phosphorylation of STAT family members. These findings suggest a strong involvement of miRNA-regulated genes in inflammation signaling and metabolic homeostasis.

Regarding cellular localization, genes associated with VLDLs and chylomicrons were significantly enriched, reinforcing the role of miR-29a and miR-122 in lipid transport and metabolism. In the molecular function category, enriched terms included cytokine binding, hormone receptor activity, and phospholipase activity, further supporting their involvement in metabolic and immune modulation.

Transcription factor enrichment (Figure 3B) revealed significant associations with PPAR DR1 Q2, STAT4, and HNF4. These TFs are well-known regulators of genes involved in lipid metabolism, insulin sensitivity, and inflammatory signaling. Importantly, many of the predicted targets of miR-29a and miR-122 are transcriptionally controlled by these TFs, indicating that the same genes may be regulated at both transcriptional and post-transcriptional levels. This convergence suggests that miR-29a and miR-122 participate in broader regulatory circuits coordinated by PPAR-, STAT-, and HNF-mediated pathways, reinforcing their central role in metabolic dysfunction relevant to MASLD and MetS.

Importantly, the disease-association map (Figure 3C) was used not to re-identify the primary diseases already listed in Table 1, but to characterize the broader pathological context of the miRNA-regulated targets. This analysis demonstrated that many of the miRNA-regulated genes are linked to MASLD, T2DM, insulin resistance, hypertriglyc-eridemia, and other metabolic and neurodegenerative diseases. These results reinforce the hypothesis that miR-29a and miR-122 are central modulators of genes governing both hepatic and systemic metabolic dysfunctions.

**Figure 4:**
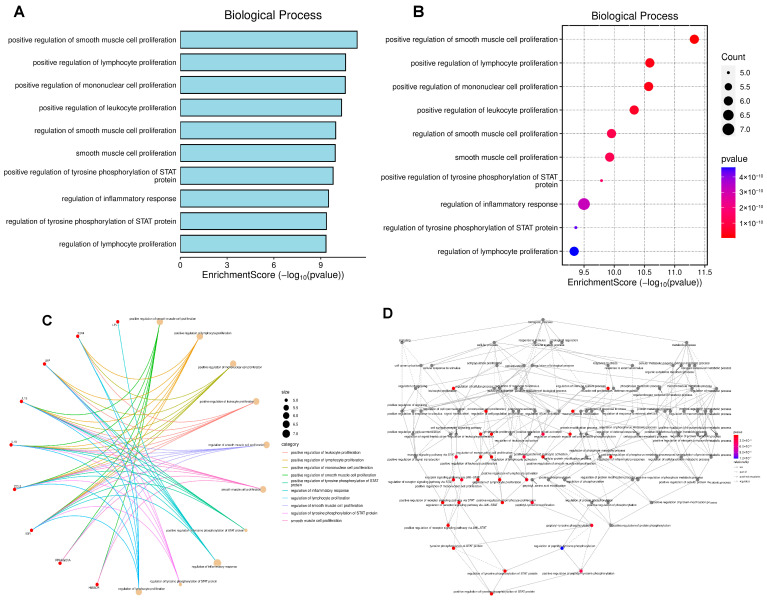
Functional enrichment analysis of genes regulated by microRNA-29a and microRNA-122. The panels display (**A**,**B**) the main enriched biological processes, (**C**) the interaction network between genes and biological processes, and (**D**) the topological map of functional interactions, highlighting the participation of genes in metabolic homeostasis, cell proliferation, and inflammatory response. (**B**,**D**) Colors indicate the intensity of the correlation, with values close to 4 × 10^−10^ (blue) indicating strong and positive correlations, values close to 1 × 10^−10^ (red) indicating strong and negative correlations, and intermediate values (pink) indicating weak or no correlation.

Figure 4 highlights the key biological processes enriched among the predicted targets of miR-29a and miR-122. The predominant themes emerging from the enrichment analysis include immune-cell proliferation, STAT signaling, inflammatory response, and pathways regulating metabolic homeostasis (Figure 4A,B). Together, these processes form highly interconnected functional clusters involving lymphocyte activation, smooth muscle cell proliferation, and cytokine-driven inflammatory signaling (Figure 4C). Network topology further demonstrates that inflammatory and metabolic regulatory pathways operate as central hubs in the functional landscape influenced by these miRNAs, indicating their potential role as upstream modulators of MetS and MASLD progression (Figure 4D).

**Figure 5:**
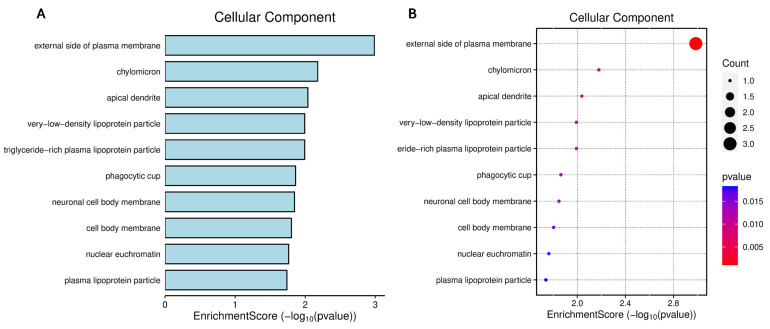

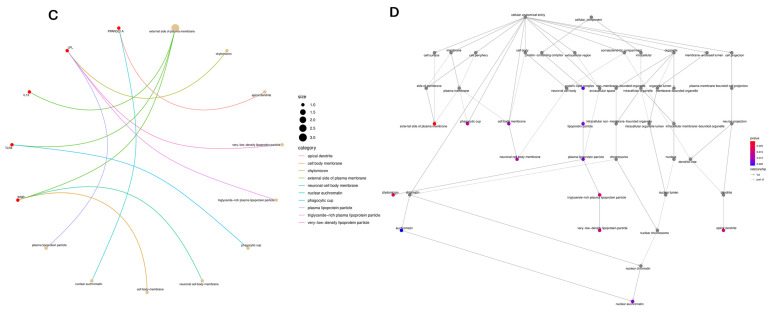
Functional enrichment analysis of cellular components associated with metabolic regulation and lipid transport is regulated by microRNA-29a and microRNA-122. Panel (**A**) displays the top enriched terms, and Panel (**B**) shows the statistical significance and number of genes involved. Panel (**C**) illustrates the gene–cellular component interaction network, and Panel (**D**) shows the functional hierarchy of the enriched terms. (**B**,**D**) Colors indicate the intensity of the correlation, with values close to 4 × 10^−10^ (blue) indicating strong and positive correlations, values close to 1 × 10^−10^ (red) indicating strong and negative correlations, and intermediate values (pink) indicating weak or no correlation.

Figure 5 highlights that the cellular components enriched among miR-29a and miR-122 targets converge primarily on membrane-associated structures involved in lipid transport and metabolic signaling. The strongest enrichment signals correspond to the external plasma membrane, lipoprotein particles, and vesicular compartments, indicating that many of the regulated genes encode proteins located at the cell surface or in lipid-handling organelles (Figure 5A,B). Functional network mapping further demonstrates that these components form interconnected clusters, suggesting coordinated regulation of membrane dynamics and lipid trafficking (Figure 5C). Topological organization of these terms reveals that membrane domains and lipoprotein particles occupy central positions within the enrichment landscape, underscoring their biological relevance to metabolic dysregulation associated with MASLD and MetS (Figure 5D).

**Figure 6:**
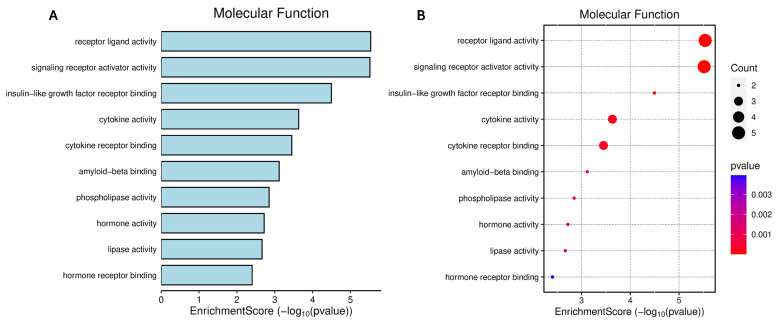

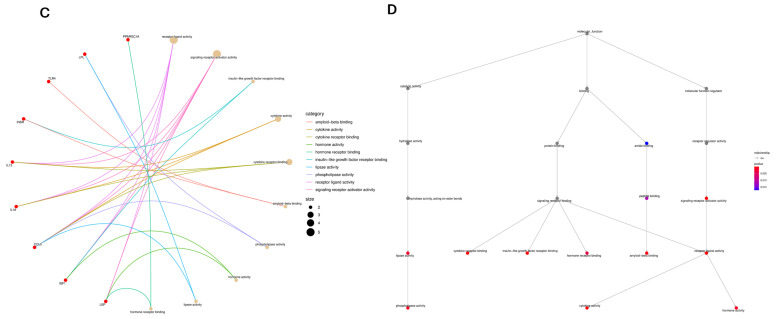
Functional enrichment analysis of genes regulated by mircoRNA-29a and microRNA-122 according to molecular function. Panel (**A**) displays the main enriched terms, and Panel (**B**) shows the statistical significance and number of genes involved. Panel (**C**) illustrates the gene–molecular function interaction network, and Panel (**D**) shows the functional hierarchy of the enriched terms. (**B**,**D**) Colors indicate the intensity of the correlation, with values close to 4 × 10^−10^ (blue) indicating strong and positive correlations, values close to 1 × 10^−10^ (red) indicating strong and negative correlations, and intermediate values (pink) indicating weak or no correlation.

Figure 6 highlights that the molecular functions enriched among miR-29a and miR-122 targets converge on receptor-mediated signaling, cytokine activity, and ligand–receptor interactions—processes central to metabolic and inflammatory regulation. The strongest signals are related to receptor ligand activity, signaling receptor activation, cytokine binding, and insulin-like growth factor interactions, revealing that these miRNAs modulate pathways crucial for intercellular communication and hormonal control (Figure 6A,B). Functional network mapping demonstrates that cytokine signaling, lipid-related signaling, and hormone-receptor engagement form interconnected modules, suggesting coordinated regulation of systemic and hepatic signaling axes (Figure 6C). Hierarchical organization of molecular functions further reveals tightly clustered pathways related to signal transduction and receptor activity, reinforcing their relevance to the progression of metabolic dysfunction in MASLD and MetS (Figure 6D).

**Figure 7:**
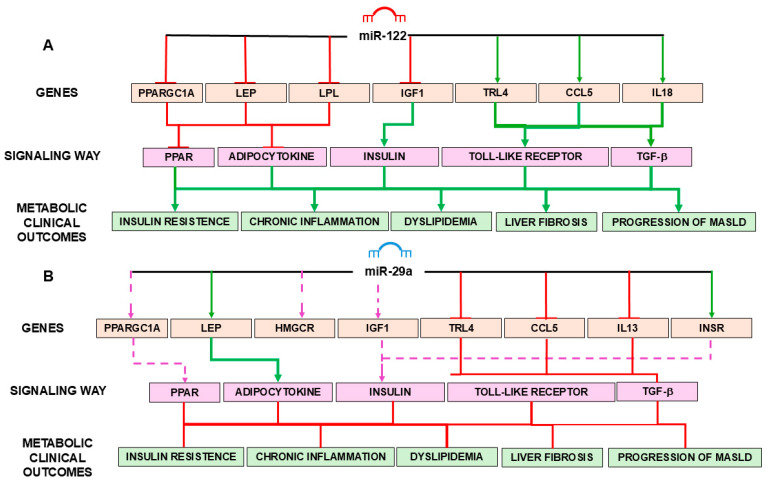
Integrated microRNA–gene–pathway interaction networks illustrating the regulatory roles of miR-122 and miR-29a in metabolic dysfunction-associated steatotic liver disease (MASLD) and metabolic syndrome (MetS). (**A**) Network depicting the regulatory effects of miR-122 on key metabolic and inflammatory genes (*PPARGC1A*, *LEP*, *LPL*, *IGF1*, *TLR4*, *CCL5*, and *IL18*) and their associated signaling pathways, including PPAR, adipocytokine, insulin, Toll-like receptor, and TGF-β pathways. The downstream metabolic and clinical outcomes include insulin resistance, chronic inflammation, dyslipidemia, liver fibrosis, and progression of MASLD. (**B**) Network illustrating the regulatory role of miR-29a on genes related to lipid metabolism, insulin signaling, and inflammation (*PPARGC1A*, *LEP*, *HMGCR*, *IGF1*, *TLR4*, *CCL5*, *IL13*, and *INSR*), highlighting its modulatory effects on PPAR, adipocytokine, insulin, Toll-like receptor, and *TGF-β* signaling pathways and associated metabolic outcomes. In both panels, green arrows indicate activation, red arrows indicate inhibition, and purple dashed arrows represent regulatory interactions. Target genes and pathways were identified based on KEGG enrichment analysis and functional annotation.

A comprehensive interaction diagram was constructed based on functional enrichment analysis of KEGG pathways regulated by the target genes of miR-122 and miR-29a (Figure 7). The diagram illustrates the molecular crosstalk between miRNAs, their validated target genes, and the metabolic and inflammatory pathways central to the pathophysiology of MASLD and MetS.

Figure 7 illustrates the molecular mechanisms mediated by miR-122 (Figure 7A) and miR-29a (Figure 7B) in the regulation of genes associated with insulin resistance, chronic inflammation, dyslipidemia, liver fibrosis, and MASLD progression. As depicted in Figure 7A, miR-122 negatively regulates essential genes such as *PPARGC1A*, *LEP*, *LPL*, and *IGF1*, directly impacting the metabolic pathways of PPAR, adipokines, and insulin signaling. Furthermore, miR-122 has an inhibitory effect on *TLR4* and *CCL5*, and acts indirectly on TGF-β, all of which are important in the activation of inflammatory and fibrogenic pathways. The combined positive and negative effects promote metabolic dysregulations that favor insulin resistance, chronic inflammation, and lipid dysfunction, culminating in liver fibrosis and MASLD progression.

On the other hand, miR-29a (Figure 7B) acts predominantly in a protective manner. It inhibits the expression of genes such as *TLR4*, *CCL5*, *IL13*, and *TGFB1*, all involved in pro-inflammatory and fibrogenesis pathways. Additionally, miR-29a has a regulatory effect on *HMGCR*, contributing to the reduction of hepatic lipogenesis, and on *INSR* and *IGF1*, favoring insulin sensitivity. The result is an attenuation of the pathological processes associated with MASLD, indicating the therapeutic potential of miR-29a as an epigenetic target.

Figure 7 therefore highlights the duality between the pro-disease effects of miR-122 and the anti-disease effects of miR-29a, supporting the idea that epigenetic manipulation via miRNA mimics is a promising approach to prevent or treat MASLD.

**Figure A1:**
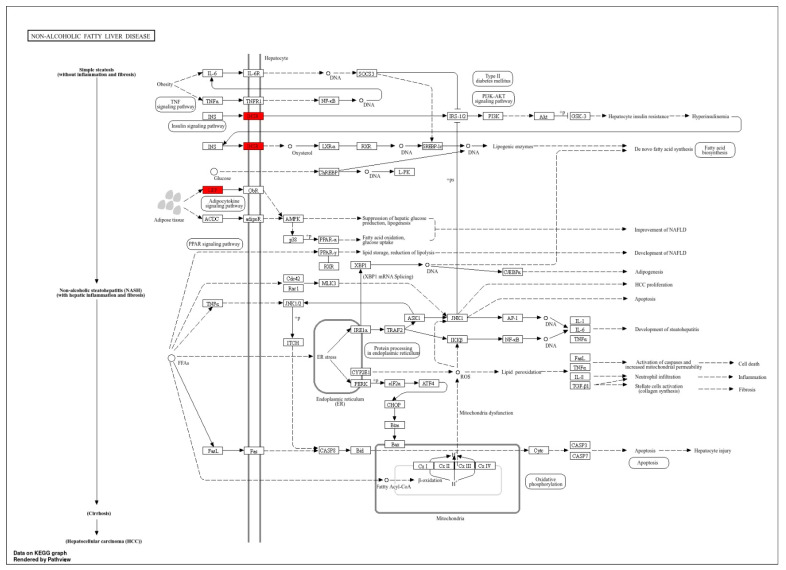
Molecular Mechanisms of Non-Alcoholic Fatty Liver Disease (NAFLD) Progression. KEGG-based pathway map illustrating molecular mechanisms involved in the progression of metabolic dysfunction-associated steatotic liver disease (MASLD). The diagram depicts key signaling pathways associated with insulin resistance, lipid metabolism, inflammation, oxidative stress, endoplasmic reticulum stress, fibrosis, and hepatocellular carcinoma development. Highlighted nodes indicate genes identified in the present analysis. Solid arrows represent direct signaling or activation, whereas dashed arrows indicate indirect or regulatory interactions. The pathway was rendered using the Pathview tool based on KEGG annotations.

**Figure A2:**
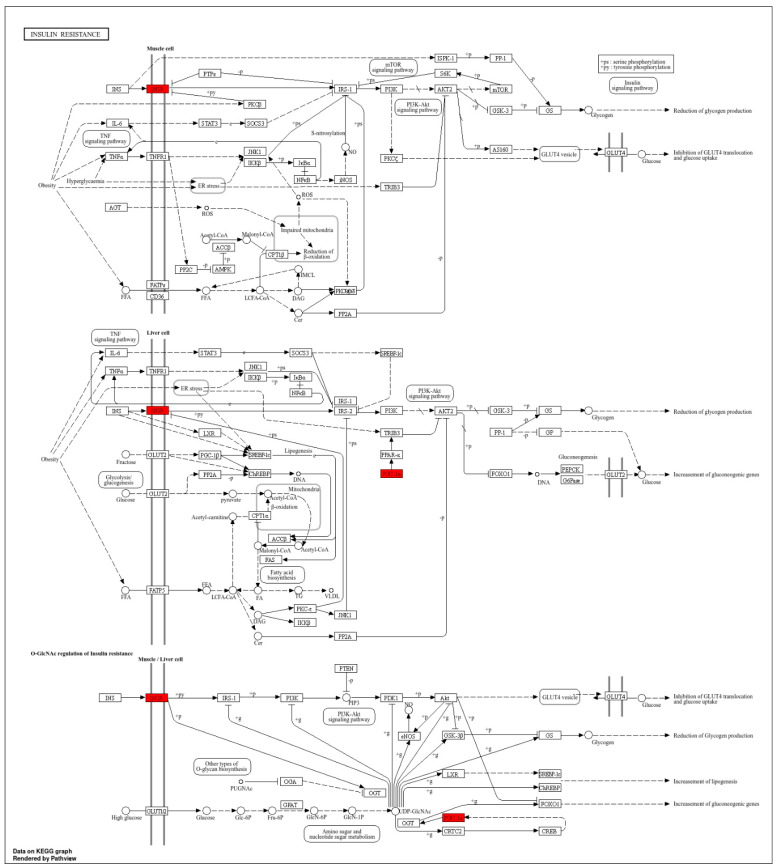
Molecular mechanisms underlying insulin resistance based on KEGG pathway analysis. The diagram illustrates key signaling events involved in the development of insulin resistance in metabolic tissues, including insulin receptor signaling, PI3K–AKT pathway modulation, glucose uptake, gluconeogenesis, lipid metabolism, oxidative stress, and mitochondrial dysfunction. The pathway highlights interactions among insulin signaling components, inflammatory mediators, and metabolic regulators contributing to impaired glucose homeostasis. Red-highlighted nodes indicate genes identified as relevant in the present analysis. Solid arrows represent direct signaling or activation, whereas dashed arrows indicate indirect or regulatory interactions. The pathway was generated using KEGG annotations and rendered with the Pathview tool.

The individual KEGG pathways related to insulin resistance and MASLD are depicted in Figure A1 and Figure A2 (Appendix A), respectively. The diagrams provide additional detail on how the predicted target genes are involved in canonical signaling cascades associated with metabolic regulation and liver dysfunction.

## 4. Discussion

Insulin resistance, a central mechanism in MetS, is critically involved in MASLD pathogenesis, exacerbating dyslipidemia, chronic inflammation, and liver injury. Notably, approximately 70% of patients with T2DM exhibit some degree of MASLD, reinforcing the notion that MetS is a major driver of liver disease [25].

The miRNAs are increasingly recognized as promising diagnostic and therapeutic biomarkers. miR-34a is implicated in mitochondrial stress and apoptosis in liver dysfunction. Concurrently, the expression of genes such as *INS*, *INSR*, *PPARG*, and *APOB* underscores the central role of insulin signaling and lipid transport in MASLD pathophysiology [26,27].

The intersection of MASLD, MetS, and T2DM reflects shared disruptions in glycolipid metabolism, which compromises liver function and heightens the risk of cardiovascular disease, systemic inflammation, and endothelial dysfunction. The relationship with genes such as *MTTP* and *LPL*, involved in lipoprotein assembly and lipid trafficking, suggests that impaired lipid homeostasis contributes to hepatic steatosis and fibrosis. These findings (Figure 1) underscore the importance of insulin signaling and lipid metabolism as critical biological pathways and potential therapeutic targets in MASLD and MetS [28,29].

The close association between MASLD and HCC suggests a potential progression from steatosis to non-alcoholic steatohepatitis (NASH), advanced fibrosis, and malignancy, particularly in individuals with obesity and MetS [30]. Additionally, the link with bone diseases, such as osteopenia and osteonecrosis, highlights the systemic nature of MASLD, likely mediated by endocrine imbalances, inflammation, and insulin resistance [31].

Taken together (Figure 1 and Figure 2), these findings highlight miR-122 and miR-29a as critical regulators of key pathogenic processes in MASLD, suggesting their potential as diagnostic and therapeutic targets. miR-122 is the most abundant hepatic miRNA and plays a central role in the regulation of lipid metabolism and hepatic homeostasis. It controls triglyceride synthesis and lipid droplet formation, processes directly implicated in hepatic steatosis. Inhibition of miR-122 was found to reduce lipid accumulation by targeting factors such as Yin Yang 1 (YY1) and the FXR/SHP signaling pathway, which are essential for hepatic lipid regulation [32]. Furthermore, miR-122 contributes to hepatic lipogenesis by repressing the LKB1/AMPK pathway through SIRT1 modulation, thereby promoting lipid deposition in the liver [33,34]. This increase in lipogenesis is associated with the development of insulin resistance, a hallmark of MASLD and MetS.

Clinically, elevated circulating levels of miR-122 correlate with dyslipidemia and insulin resistance in individuals with MetS and T2DM, suggesting its potential as a biomarker for cardiometabolic risk [13]. Additionally, miR-122 influences glucose homeostasis and insulin sensitivity, reinforcing its relevance in the pathophysiological progression of MASLD. In addition to metabolic regulation, miR-122 influences hepatic inflammation and autophagy. It regulates pro-inflammatory mediators, such as PKM2, iNOS, and COX2, proteins implicated in the inflammatory cascade of MASLD [26,35]. It also participates in cardiovascular complications through the miR-122/Sirt6/ACE2 pathway, which links hepatic and systemic metabolic stress [27,35].

Conversely, miR-29a exhibits a hepatoprotective profile. It is involved in key mechanisms that alleviate mitochondrial stress and hepatic inflammation, crucial factors in the transition from MASLD to NASH. miR-29a modulates mitochondrial proteostasis by regulating the unfolded protein response and inhibiting GSK3β, a kinase associated with mitochondrial stress [36]. It also promotes mitochondrial biogenesis via SIRT1 activation, reducing oxidative stress and fibrotic progression [36,37,38].

miR-29a plays a vital role in liver lipid homeostasis by suppressing lipoprotein lipase (LPL), thereby limiting excessive lipid uptake by hepatocytes and reducing the risk of steatosis [37]. Its antifibrotic effects are evidenced by the downregulation of profibrotic and pro-inflammatory genes, decreasing extracellular matrix deposition and inhibiting hepatic stellate cell activation [39]. Collectively, these findings support the therapeutic potential of miR-122 and miR-29a in mitigating MASLD progression and its metabolic complications.

Whereas the bibliometric review primarily mapped research trends and highlighted the diagnostic and therapeutic interest in miR-122 and miR-29a, the present in silico analysis advances this knowledge by elucidating their predicted gene networks, enriched pathways, and functional interactions, thus providing mechanistic insight underlying their recurrent citation in MASLD literature [40].

In addition to miR-122 and miR-29a, other miRNAs have emerged as relevant players in the context of inflammation, insulin resistance, and MASLD. For example, miR-21, although less abundant in liver tissues, has been consistently found to be overexpressed in MASLD and is associated with inflammation, fibrosis, and hepatocarcinogenesis [41,42,43]. Its pathological effects are attributed to repression of PPARα, a central regulator of lipid metabolism and hepatic inflammation [29,43].

Another example is miR-34a. Commonly overexpressed under metabolic stress, this miRNA suppresses HNF4α, a nuclear receptor critical for lipoprotein metabolism. miR-34a inhibition disrupts VLDL secretion, promoting triglyceride accumulation and progression of steatosis [28,44,45,46].

Also noteworthy are miR-126, miR-155, miR-192, miR-193, miR-222, and miR-223. miR-126 has been implicated in insulin resistance through suppression of IRS-1, a key protein in insulin signaling. Additionally, it contributes to hepatic fibrosis via activation of hepatic stellate cells [47,48,49].

miR-155 is a pro-inflammatory miRNA whose deficiency reduces hepatic lipid accumulation. Overexpression of miR-155 and NF-κB correlates with mitochondrial dysfunction and oxidative stress in MASLD, as evidenced by increased reactive oxygen species generation, mitochondrial damage, and disrupted energy metabolism [50,51,52].

The role of miR-192 in MASLD remains controversial. Some studies associated its overexpression with enhanced lipid accumulation and YY1-mediated lipid metabolism disruption, whereas other investigations suggested that its suppression exacerbates steatosis. Its pro-inflammatory role is linked to increased IL-6 and TNF-α expression [53,54].

As for miR-193, it targets PGC1α, a transcriptional coactivator involved in mitochondrial function and energy metabolism. Its inhibition compromises fatty acid oxidation and glucose metabolism, contributing to steatotic changes in prediabetic individuals [55].

miR-222 has been described as a promising biomarker of liver fibrosis and HCC, showing high sensitivity and specificity. It is overexpressed in cirrhotic and neoplastic tissues and contributes to tumor progression by modulating BBC3, Bcl-2, cyclin D1, and caspase-3 expression [56,57,58]. Moreover, miR-222 promotes fibrogenesis by activating LX-2 cells and increasing α-SMA and collagen I expression. Its upregulation in biliary atresia models is mediated by NF-κB signaling and associated with cholestasis and inflammatory damage [59,60].

Finally, miR-223 is known for its anti-inflammatory and antifibrotic roles. It is secreted in extracellular vesicles by neutrophils and delivered to hepatocytes via LDLR, especially in the presence of free fatty acids. Once internalized, miR-223 reduces the expression of inflammatory and fibrotic genes, limiting NASH progression [61,62]. Furthermore, IL-6 signaling enhances the production of miR-223-enriched exosomes, which downregulate TAZ expression and attenuate liver fibrosis [63]. In murine models, miR-223 deletion accelerates NASH development and increases pro-inflammatory gene expression, confirming its protective role in liver disease [64].

Previous studies have shown that miR-29a and miR-122 are among the miRNAs most frequently associated with chronic liver diseases, including NAFLD, NASH, and decompensated cirrhosis [65,66,67]. However, most of these investigations focus on the differential expression of miRNAs in biological samples, without delving into the functional identification of their target genes. In a recent study, integrative analysis using bioinformatics predictions (miRGate) and experimental validation revealed that miR-29a and miR-122 regulate genes such as *BCL2* and *XIAP*, involved in apoptosis and hypoxic stress response pathways, indicating a relevant functional role in the progression of liver injury [66].

Collectively, these findings suggest that the gene targets of miR-29a and miR-122 are predominantly located in structures essential for lipid trafficking, signal transduction, and metabolic regulation. This spatial distribution supports the hypothesis that dysregulation of these miRNAs could impair membrane-bound receptor signaling, lipoprotein metabolism, and cellular communication, contributing to the pathogenesis of MASLD and MetS.

The findings (Figure 3) underscore the involvement of miR-29a and miR-122 in modulating genes that govern molecular recognition events, cell surface signaling, and endocrine regulation. Such processes are crucial for metabolic homeostasis and inflammatory responses implicated in MASLD pathogenesis.

The combined results from Figure 4, Figure 5 and Figure 6 underscore the pivotal roles of miR-29a and miR-122 in modulating genes involved in critical metabolic, inflammatory, and signaling pathways. These miRNAs influence a broad spectrum of biological processes ranging from cell proliferation and immune response to lipid transport and receptor-mediated signaling, as evidenced by the enriched categories across biological processes, cellular components, and molecular functions.

This integrative functional landscape highlights that miR-29a and miR-122, through their tightly regulated gene networks, may serve as both non-invasive biomarkers and therapeutic targets of metabolic disorders, in particular MetS and MASLD.

miR-122, predominantly expressed in hepatocytes, negatively regulates several key metabolic genes, including *PPARGC1A*, *LPL*, *HMGCR*, *IGF1*, and *TLR4*. These interactions converge on critical signaling hubs, such as the PPAR, insulin, adipocytokine, and TLR pathways. The downstream consequences of miR-122 activity include increased insulin resistance, dyslipidemia, chronic inflammation, and fibrogenic remodeling, which contribute to MASLD progression.

By contrast, miR-29a appears to exert protective effects, modulating targets such as *LEP*, *INSR*, *CCL5*, *IL13*, and *IL18*. Its regulatory activity enhances insulin sensitivity, attenuates pro-inflammatory signaling, and suppresses TGF-β-mediated fibrogenesis, suggesting a hepatoprotective role.

The findings reinforce the divergent functional roles of the miRNAs: whereas miR-122 is associated with the promotion of metabolic and inflammatory dysfunction, miR-29a is a potential therapeutic target with anti-inflammatory and anti-fibrotic effects. The miRNAs represent important biomarkers and strategic points of intervention in the prevention and management of MASLD and its comorbidities.

The findings of this bioinformatics analysis show the importance of miR-122 and miR-29a as key regulators in the pathophysiology of MASLD. The analysis allowed identifying target genes and metabolic pathways associated with insulin resistance, chronic inflammation, and mitochondrial dysfunction, central mechanisms in both MASLD progression and systemic MetS alterations. miR-122, expressed mainly in the liver, has been shown to be involved in triglyceride accumulation, modulation of inflammatory pathways, and regulation of hepatic autophagy. miR-29a exerts hepatoprotective effects, with an important role in reducing mitochondrial stress and suppressing hepatic fibrosis through the regulation of genes such as *GSK3B*, *SIRT1*, and *LPL*. The overlap of target genes implicated in MASLD, MetS, and T2DM, such as *PPARGC1A*, *TLR4*, *LEP*, *IL13*, and *INSR*, reveals the connection between these metabolic conditions.

Functional enrichment analysis also demonstrated the participation of genes in biological pathways linked to lipoprotein regulation, inflammation, and glucose and lipid metabolism. These results support the hypothesis that epigenetic regulation mediated by miRNAs is a key element in liver homeostasis and may represent a promising avenue for the development of therapeutic strategies. The data underscore the potential of these miRNAs as non-invasive biomarkers and emerging therapeutic targets, capable of contributing to early diagnosis and the development of more effective therapeutic approaches.

A deeper understanding of the molecular pathways modulated by these miRNAs may contribute to the development of personalized interventions in complex metabolic diseases. However, future experimental and clinical studies are needed to validate the targets identified in silico and evaluate the clinical applicability of miRNA modulation in the prevention and treatment of MASLD.

## 5. Conclusions

This bioinformatics analysis demonstrated that miR-122 and miR-29a play central and functionally divergent roles in the regulation of metabolic and inflammatory pathways involved in the pathophysiology of MASLD. miR-122 was associated with dysregulated lipid metabolism, insulin resistance, and hepatic inflammation by negatively modulating key genes, such as *PPARGC1A*, *PPARA*, *LPL*, *IGF1*, *TLR4*, and *HMGCR*, which participate in critical pathways including PPAR, insulin signaling, and inflammatory cascades. On the other hand, miR-29a exhibited hepatoprotective effects, with potential anti-inflammatory and antifibrotic properties. It positively regulates genes, such as *LEP*, *INSR*, *CCL5*, *IL13*, and *IL18*, contributing to improved insulin sensitivity and attenuation of liver fibrosis.

Functional enrichment analysis revealed that genes regulated by these miRNAs are significantly involved in lipid homeostasis, STAT phosphorylation, inflammatory response, and cell proliferation, as well as being associated with cellular structures such as lipoproteins and plasma membranes.

These findings underscore the potential of miR-122 and miR-29a as non-invasive biomarkers and emerging therapeutic targets in complex metabolic conditions, particularly in MASLD associated with MetS. Although promising, their roles must be validated through in vitro, in vivo, and clinical studies to confirm the identified molecular targets and biological pathways. The translational potential of miRNAs as non-invasive biomarkers and therapeutic targets is noteworthy, with possible applications in early diagnosis and personalized medicine; however, their clinical use still requires further investigation. This study, based on in silico analyses, has inherent limitations of computational prediction, reinforcing the need for integrated approaches that combine bioinformatics, clinical, and experimental data to achieve a more comprehensive understanding of MASLD pathophysiology.

## Figures and Tables

**Table 1:** Target genes of microRNAs-29a and -122 associated with type 2 diabetes mellitus (T2DM), metabolic dysfunction-associated steatotic liver disease (MASLD), and metabolic syndrome (MetS).

microRNA	Genes Regulated in T2DM	Genes Regulated in MASLD	Genes Regulated in MetS
29a-3p	***n* = 13** (***VDR***, ***HMGCR***, *CRSB*, *IL33*, *CLOCK*, *PRAM1*, *SLC2A3*, *AQP4*+, *KCNJ11*, ***PPARGC1A*+**, ***GSR***, *SERPINE1*, *BMP8B*)	***n* = 14** (***VDR***, ***HMGCR***, *STAT3*, *SOD2*+, ***LEP***, ***PPARGC1A*+**, *ALDH1L2*, *ALDH2*+, *PNPLA3*, *ACO1*, *SYRPINE1*, ***GSR***, *IRS1*, *SIDT2*)	***n* = 2** (***LEP*+**, *T53INP1*+)
29a-5p	***n* = 21** (***CCL5***, ***PPRGC1A*+**, *F7*+, *INS*, *LRP1*, *PTPRN2*, ***TLR4*+**, *AQP4*+, ***INSR*+**, ***IGF1*+**, *PTPRN2*+, *UCP3*, ***VDR***, *CASP3*, ***HMGCR***, *CLOCK*+, *PTEN*+, *HGF*, *SRD5A2*, *NROB2*, *ICAM1*)	***n* = 15** (*IL18*+, ***CCL5*+, *PPARGC1A*+**, *ALDH2*+, *DGAT2*, *JAK2*+, *PGRMC1*+, *IL13*+, ***TLR4*+**, ***INSR***, ***IGF1*+**, ***LEP*+**, *SOD2*+, ***VDR***, ***HMGCR*)**	***n* = 1** (***LEP*+**)
122-3p	***n* = 7** (***MTHFR***, *ITGA2* *, *TLR4*+, *GAD1* *, *GIPR*, ***GSR***, *BNP8B*)	***n* = 11** (***MTHFR***, *ADIPOQ*, *PPARAGC1A* *+, *TLR4* *+, *CYP8B1*, *STAT3*, *SOD2* *+, *ALDH2* *, *SLC7A3*, *ADRB3*, ***GSR***)	***n* = 0**
122-5p	***n* = 23** (***PPARGC1A*+**, *F7*+, *ABCG2*, *CLOCK*+, *SLC2A3*, *GAD1* *, *ITGA2* *, *PTPRN2*+, ***CCL5***, ***TLR4*+**, *HFE*, *MAX*, *PTEN*+, *IL2RA*, *AQP4*+, *CYP11B3*, *GGT1*, *ICA1*, *F3*, *INSR*+, ***IGF1*+**, *CTSB*, *UP3*)	***n* = 34** (*IDE*, *PPARGC1A* *+, *IL1RN*, *CIDEB*, *NR1H4*, *AIP5F1A*, *PGRMC1*+, *JAK2*+, ***CCL5*+**, *TRL4* *+, *IRS2*, *ATP5F1A*, *IGF1*+, *HFE*, *IGF1R*, *TFRC*, *SOD2* *+, ***LEP*+**, *LEPR*, *ACO1*, *CD163*, *IL18*+, *ALDH2* *+, *PGRMC1*+, *ABCG8*, *IL13***+**, *MFN2*, *GPT2*, *IL1B*, *NR1H4*, *DGAT2*, *CYP8B1*, *ADRB3*, **LPL**)	***n* = 3** (***LEP*+**, *T53INP1*+, ***LPL***)

Genes in bold appear in more than one disease category while being regulated by the same microRNA. Underlined genes represent those that occur multiple times within the same disease category and microRNA strand. An asterisk (*) indicates genes targeted by both miR-29 and miR-122. A plus sign (+) denotes genes appearing in the same disease category but under different microRNAs (e.g., 29a-3p vs. 29a-5p).

## Data Availability

FAPESC, Public Call No. 21/2024, Grant Agreement No. 2024TR002215.

## Abbreviations

The following abbreviations are used in this manuscript:

MASLD	Metabolic dysfunction-associated steatotic liver disease
MetS	Metabolic Syndrome
HCC	Hepatocellular carcinoma
miRNAs	MicroRNAs
CDS	Coding DNA sequence
KEGG	Kyoto Encyclopedia of Genes and Genomes
FDR	False discovery rate
T2DM	Type 2 diabetes mellitus
NASH	Non-alcoholic steatohepatitis
YY1	Yin Yang 1
LPL	Lipoprotein lipase

## Appendix A

### Appendix A.1. Figure A1

Figure A1—Molecular Mechanisms of Non-Alcoholic Fatty Liver Disease (NAFLD) Progression. The schematic pathway illustrates the complex molecular network underlying the pathogenesis and progression of non-alcoholic fatty liver disease (NAFLD), from simple steatosis to non-alcoholic steatohepatitis (NASH), fibrosis, cirrhosis, and potentially hepatocellular carcinoma (HCC). The diagram integrates signaling cascades triggered by metabolic overload, inflammation, lipotoxicity, and cellular stress, particularly within hepatocytes and adipose tissue.

Key highlighted pathways include the following: Insulin signaling pathway: Dysregulation of insulin signaling (INS –> IRS –> PI3K –> Akt) contributes to hepatic insulin resistance, lipogenesis, and hyperinsulinemia, with key disruptions involving IRS1 and IRS2 (highlighted in red). These defects impair glucose metabolism and promote fatty acid synthesis. Inflammatory signaling: Obesity-induced cytokines, such as TNF-α and IL-6, activate NF-κB and JNK pathways, promoting hepatic inflammation and insulin resistance. IL-6–mediated SOCS3 expression further exacerbates insulin signaling inhibition. Adipocytokine signaling: Leptin (LEP) and adiponectin modulate hepatic metabolism via AMPK and PPARα/γ pathways. In NAFLD, decreased adiponectin and increased leptin lead to impaired fatty acid oxidation and enhanced lipogenesis. PPAR signaling pathway: Activation of PPARα and PPARγ promotes lipid storage and glucose uptake, whereas downregulation contributes to hepatic lipid accumulation and oxidative stress. ER stress and mitochondrial dysfunction: Lipotoxicity-induced stress (via IRE1α, PERK, and ATF6) leads to CHOP-mediated apoptosis, mitochondrial dysfunction (increased ROS), and activation of caspase cascades (CASP3, CASP8, CASP9), promoting hepatocyte injury. Fibrosis and inflammation: Cytokines (e.g., TNF-α, IL-1, TGF-β1) stimulate hepatic stellate cells and matrix remodeling, contributing to fibrosis. Neutrophilic infiltration and sustained inflammation are central features of NASH progression. This integrated KEGG pathway underscores the multifactorial nature of NAFLD, highlighting critical molecular targets involved in insulin resistance, lipid dysregulation, inflammation, oxidative stress, and apoptosis. The red boxes in the diagram emphasize genes (e.g., IRS1, IRS2, LEP) identified in our analysis as relevant to the metabolic and inflammatory alterations observed in the study population, suggesting potential mechanisms contributing to the observed clinical and biochemical profiles.

### Appendix A.2. Figure A2

Figure A2—Molecular Mechanisms Underlying Insulin Resistance. This figure represents the KEGG pathway for insulin resistance, highlighting the intricate network of molecular interactions in muscle, liver, and adipose tissues that contribute to impaired insulin signaling a central feature in metabolic syndrome and steatotic liver disease (SLD-MD). Key components of the pathway include the followjng: Insulin receptor signaling: Insulin binding to its receptor (INSR) initiates a cascade via IRS1 and IRS2, activating PI3K and downstream targets like AKT2. In insulin-resistant states, as highlighted in red (IRS1, IRS2, PIK3R1, AKT2), defects in these proteins impair glucose uptake (via GLUT4) and suppress glycogen synthesis and lipogenesis. Inflammatory and cytokine signaling: TNF-α and IL-6, elevated in obesity and hepatic steatosis, activate pathways (e.g., JNK, SOCS3, NF-κB) that inhibit IRS1/2 phosphorylation, reducing insulin sensitivity. These events link chronic inflammation to metabolic dysregulation, reinforcing the concept of metaflammation. Lipid intermediates and mitochondrial dysfunction: Accumulation of diacylglycerol (DAG), ceramides, and oxidative stress in mitochondria (via ROS) disrupts insulin signaling by activating kinases like PKCθ, further impairing AKT activation. Glucose and lipid metabolism: In the liver, impaired PI3K-AKT signaling increases expression of gluconeogenic genes (e.g., PEPCK, G6PC) and reduces glycogen synthesis, contributing to hyperglycemia. The O-GlcNAc modification pathway (OGT/OGA) also regulates insulin sensitivity and is dysregulated in insulin resistance, as seen by changes in OGT and SLC25A4 (highlighted). Adipose tissue and GLUT4 translocation: In adipocytes, reduced GLUT4 expression and vesicle trafficking impair glucose uptake, a hallmark of systemic insulin resistance. The genes highlighted in red (IRS1, IRS2, AKT2, PIK3R1, OGT, SLC25A4) were identified in the study as differentially expressed or potentially dysregulated in individuals with steatosis and metabolic syndrome, corroborating their central role in insulin resistance. Taken together, this pathway reinforces the concept that insulin resistance arises from a convergence of inflammatory, metabolic, and mitochondrial stress signals, affecting multiple tissues. It highlights potential molecular targets for early diagnosis and intervention in metabolic liver disease.
